# Trefoil factor family proteins as potential diagnostic markers for mucinous invasive ovarian carcinoma

**DOI:** 10.3389/fonc.2022.1112152

**Published:** 2023-02-02

**Authors:** Elisabeth Werner Rönnerman, Daniella Pettersson, Szilárd Nemes, Pernilla Dahm-Kähler, Anikó Kovács, Per Karlsson, Toshima Z. Parris, Khalil Helou

**Affiliations:** ^1^ Department of Oncology, Institute of Clinical Sciences, Sahlgrenska Academy, University of Gothenburg, Gothenburg, Sweden; ^2^ Sahlgrenska Center for Cancer Research, Sahlgrenska Academy, University of Gothenburg, Gothenburg, Sweden; ^3^ Department of Clinical Pathology, Sahlgrenska University Hospital, Gothenburg, Sweden; ^4^ Department of Orthopedics, Institute of Clinical Sciences, Sahlgrenska Academy, University of Gothenburg, Gothenburg, Sweden; ^5^ Department of Obstetrics and Gynecology, Institute of Clinical Sciences, Sahlgrenska Academy, University of Gothenburg, Gothenburg, Sweden

**Keywords:** mucinous ovarian carcinoma, molecular classification, trefoil factor gene family, histotype, biomarkers

## Abstract

**Introduction:**

Ovarian cancer (OC) is the leading cause of gynecological cancer-related death. Of the main OC histologic subtypes, invasive mucinous carcinomas (MC) account for only 3% of OC cases and are frequently associated with favorable prognosis. Nevertheless, MCs differ greatly from the other OC histotypes in clinical, pathological, and biological behavior. However, the origin and molecular pathogenesis of MC are not yet fully understood. Therefore, identification of novel diagnostic markers could potentially facilitate early diagnosis of OC, particularly the MC histotype, thereby leading to the development of histotype-specific treatment regimens and improved survival rates.

**Methods:**

In the present study, Trefoil factor gene family members (TFF1, TFF2 and TFF3) were identified as MC histotype-specific biomarkers using RNA sequencing (RNA-seq) data for 95 stage I-II OCs. The diagnostic value of TFF1, TFF2 and TFF3 was then evaluated by immunohistochemistry on 206 stage I-II OCs stratified by histotype (high-grade serous carcinoma [HGSC], endometrioid carcinoma [EC], clear cell carcinoma [CCC], and MC).

**Results:**

We showed significantly elevated intracytoplasmic protein expression levels for TFF1, TFF2 and TFF3 in MC samples, thereby revealing an association between expression of Trefoil factor gene family members and the MC histotype. Taken together, these findings suggest that the TFF proteins may play a pivotal role in tumor initiation and progression for the MC histotype.

**Conclusion:**

Taken together, these findings suggest that the TFF proteins may play a pivotal role in tumor initiation and progression for the MC histotype. Moreover, these novel histotype-specific diagnostic biomarkers may not only improve patient stratification of early-stage ovarian carcinomas but may also be candidates for the development of molecular targeted therapies.

## Introduction

The multipotent cells in the ovary allow for tumor formations of a highly variable nature. However, the vast majority (>90%) of malignant ovarian tumors are of epithelial origin and designated as epithelial ovarian cancer (EOC, or simplified OC), thereby true carcinomas (1–3). According to the current World Health Organization (WHO) classification (2, 3), the five main EOC histological subtypes include high-grade serous carcinoma (HgSC), endometroid (EC), mucinous (MC) and clear cell (CCC), followed by low-grade serous carcinoma (LgSC). It is a well-established fact that the different histological subtypes of EOC are separate diseases in terms of epidemiologic risk factors, morphologic precursors, biomarker expression, genotype, responsiveness to standard treatment modalities, and clinical outcomes (4–7). EOCs have the highest mortality rate among gynecological cancers (non ASR, age-standardized rate, >60%) (8). Combined factors, *e.g.* greater awareness of symptoms, optimization of surgical procedures and disease-specific treatment regimens, strongly contributed to a delicate but notable decline in death rates, thereby improving patient clinical outcome (9).

Mucinous carcinoma is the least common epithelial histotype, with an estimated prevalence of only 3% (10). However, MCs differ in many aspects from the other OC histotypes, with respect to their molecular signatures, pathological characteristics, mode of tumor progression and clinical behavior (4–6, 10–13). The 5-year survival rates for MC patients with early-stage disease (stage I – II) receiving surgical therapy alone are excellent (90%), particularly for tumors with invasive expansile growth patterns. In contrast, the significantly lower response rate to conventional carboplatin-based therapy compared to stage III – IV OC disease confers a considerably inferior outcome for late-stage MC. Besides optimized surgery, alternative treatment options for advanced MC are fairly sparse (6, 14–17). The limited curative potential for therapeutic chemotherapy highlights the relevance of a correct primary diagnosis. When carefully weighed together with established clinical parameters (*e.g.*, tumor size, laterality, etc.), the routine morphologic evaluation of OC tissue by a pathologist still applies as the diagnostic gold standard (18, 19). Although accurate subtype classification is straightforward for an experienced pathologist, unexpected perplexities in tumor constituents still have the potential to cause debate and assessment difficulties. For these cases, immunohistochemical algorithms that were developed for several of the EOCs have greatly improved decision making in OC histotyping (20–25). However, the existing immunopanels lack reliable positive indicators for MC (6). Hence, diagnosis of MCs relies on routine morphology and/or exclusion of fulfilling criteria for other histotypes, metastatic disease or a borderline tumor. Therefore, the development of easily accessible screening tools and MC-specific diagnostic biomarkers would fulfill a long-standing need (26–29). Novel biomarkers with potential to facilitate OC subtyping could be used to develop a reliable and simple algorithm that in turn could lead to revised consensus treatment guidelines (6, 22, 25, 27, 28, 30–32), optimized selection conditions for targeted therapies (15, 32, 33) as well as avoid over treatment.

The trefoil factor family (TFF) consists of a group of three small peptides (TFF1 – 3) with a rather unique appearance comprised of a high amino acid content and disulfide bonds giving the peptide a clover leaf-like or trefoiled domain. Immunohistochemical analysis has demonstrated cytoplasmic expression of these peptides in normal gastrointestinal mucosa including goblet cells (TFF1 especially). Moreover, these expression patterns have also been observed in the mucosal lining of virtually all tissues containing mucus-secreting cells, including the respiratory tract (TFF3), ocular epithelium, prostate, and female reproductive organs like cervix uteri. Detection of TFF proteins secreted in certain body fluids such as saliva, blood, urine, and breast milk are thought to reflect para-, auto- and/or endocrine functions (34, 35). Data suggests that trefoil peptides exert multiple functions, including influence of angiogenesis, proliferation, antiapoptotic properties and differentiation. Several studies show that TFF proteins interact with oncogenic signaling pathways as well as play a role in cell migration. For example, *via* the HIF-1-alpha pathway, TFF1 is involved in mucosal repair under normal conditions and exerts auto induced EMT-like transition, which is favorable in hypoxic malignant condition (35, 36). Thus, altered TFF expression levels drives tumorigenesis (34, 35). A role in tumor progression *via* stimulation of cell migration, survival, invasiveness, and distant spread is further supported by detection of elevated levels of TFF1 in pancreatic, colonic, and ovarian tumor tissues (36, 37). Members of the TFF protein family have also been included in immunohistochemistry (IHC) algorithms for characterizing both special histotypes, *e.g.* TFF3 as a biomarker for ovarian carcinoma subtyping, as well as identifying carcinomas of unknown primary origin (CUP) (31, 38).

Here we identified genes in the TFF family as potential diagnostic biomarkers for the MC histotype using RNA sequencing data for 95 early-stage (stage I – II) OCs which were validated with immunohistochemistry for 206 early-stage OCs stratified by histotype. Consequently, the present study highlights TFF1 as a novel biomarker for the MC histotype that can easily be incorporated in routine histopathological diagnostic testing, thereby improving our understanding of MC histotype biology.

## Materials and methods

### Patients and tumor samples

Full-face formalin-fixed paraffin-embedded (FFPE) specimens were obtained from the Departments of Clinical Pathology at hospitals in Western Sweden for 206 early-stage (stage I and II, according to the International Federation of Gynecology and Obstetrics, FIGO, 2014 system) primary invasive OC patients diagnosed between 1994 and 2006, as previously described (39). Samples represented only by microinvasive disease or mucinous borderline tumors were excluded. Of the 206 cases analyzed by immunohistochemistry, 95 tumors were previously analyzed by RNA-seq as a training cohort in addition to the 111 tumors in the validation cohort. Clinical data were retrieved from the Swedish Quality Registry for Gynecological Cancer (SQRGC; Stockholm, Sweden) and the Cancer Registry at the National Board of Health and Welfare (Stockholm, Sweden; **Table 1**). Each FFPE sample was reclassified according to the current WHO histotype criteria (2, 3) with regard to histotype and histological grade by three independent board-certified pathologists with competence in gynecological pathology (EWR, AK, CM) using 4 µm full-face FFPE sections stained with hematoxylin and eosin. National treatment guidelines with contemporary protocols for standard surgery procedures (staging and adequate debulking cytoreductive surgery) were followed for all 206 patients. The study procedures were performed in accordance with the Declaration of Helsinki and approved by the Regional Ethical Review Board (Gothenburg, Sweden; S 164-02, 767-14 and T530-16). Due to the retrospective study design, the Regional Ethical Review Board approved a waiver of written consent to use the tumor specimens.

**Table 1:** Clinicopathological features for the 206 ovarian cancer patients in the training and validation cohorts.

	Number of patients (%)							
	Training cohort (n=95)				Validation cohort (n=111)			
	HGSC	EC	MC	CCC	HGSC	EC	MC	CCC
All	50 (53)	17 (18)	11 (12)	17 (18)	44 (40)	29 (26)	18 (16)	20 (18)
Patient age								
Mean	64	64	61	63	65	61	60	66
Range	32-86	25-83	39-80	42-84	22-88	29-81	30-82	51-84
Overall Survival								
0-2y	2 (4)	1 (6)	3 (27)	2 (12)	5 (11)	2 (7)	3 (17)	3 (15)
2-5y	17 (34)	5 (29)	2 (18)	3 (18)	9 (20)	4 (14)	1 (6)	7 (35)
5-10y	18 (36)	5 (29)	3 (27)	7 (41)	10 (23)	2 (7)	4 (22)	1 (5)
>10y	13 (26)	6 (35)	3 (27)	5 (29)	20 (45)	21 (72)	10 (56)	9 (45)
Cause of death								
Ovarian carcinoma	37 (74)	3 (18)	2 (18)	10 (59)	21 (48)	4 (14)	3 (17)	9 (45)
Other cancer	7 (14)	3 (18)	3 (27)	0 (0)	1 (2)	3 (10)	2 (11)	2 (10)
Other	5 (10)	6 (35)	4 (36)	6 (35)	5 (11)	4 (14)	5 (28)	1 (5)
Not available	0 (0)	0 (0)	0 (0)	1 (6)	2 (5)	1 (3)	1 (6)	0 (0)
Alive	6 (12)	5 (29)	2 (18)	0 (0)	15 (34)	17 (59)	7 (39)	8 (40)
Stage								
I	29 (58)	11 (65)	9 (82)	14 (82)	22 (50)	21 (72)	13 (72)	17 (85)
II	21 (42)	6 (35)	2 (18)	3 (18)	22 (50)	8 (28)	5 (28)	3 (15)
Tumor grade EC								
FIGO grade I	NA	2 (12)	NA	NA	NA	9 (31)	NA	NA
FIGO grade II	NA	9 (53)	NA	NA	NA	18 (62)	NA	NA
FIGO grade III	NA	6 (35)	NA	NA	NA	2 (7)	NA	NA
Dualistic model								
Type I	0 (0)	17 (100)	11 (100)	17 (100)	0 (0)	29 (100)	18 (100)	20 (100)
Type II	50 (100)	0 (0)	0 (0)	0 (0)	44 (100)	0 (0)	0 (0)	0 (0)
CA125								
<35	8 (16)	7 (41)	5 (45)	6 (35)	9 (20)	6 (21)	5 (28)	8 (40)
35-65	29 (58)	0 (0)	2 (18)	1 (6)	31 (70)	7 (24)	6 (33)	7 (35)
>65	13 (26)	10 (59)	4 (36)	10 (59)	4 (9)	15 (52)	7 (39)	5 (25)
Not available	0 (0)	0 (0)	0 (0)	0 (0)	0 (0)	1 (3)	0 (0)	0 (0)
Ploidy								
near diploid	15 (30)	7 (41)	2 (18)	1 (6)	7 (16)	10 (34)	5 (28)	4 (20)
aneuploid	35 (70)	9 (53)	8 (73)	16 (94)	34 (77)	17 (59)	11 (61)	14 (70)
Not available	0 (0)	1 (6)	1 (9)	0 (0)	3 (7)	2 (7)	2 (11)	2 (10)
Chemotherapy								
Yes	49 (98)	17 (100)	11 (100)	17 (100)	42 (95)	25 (86)	16 (89)	20 (100)
No	0 (0)	0 (0)	0 (0)	0 (0)	0 (0)	0 (0)	0 (0)	0 (0)
Not available	1 (2)	0 (0)	0 (0)	0 (0)	2 (5)	4 (14)	2 (11)	0 (0)

na, not applicable.

### Selection of histotype-specific candidate biomarkers

To identify novel biomarkers associated with specific OC histotypes, RNA sequencing (RNA-seq) data reported in our previous work (40) were re-evaluated and correlated with clinicopathologic features. In brief, a series of one vs all other logistic regression classifiers were performed for each histotype using RNA-seq FPKM (fragments per kilobase of exon per million fragments mapped) values. For each histotype, tumors belonging to the analyzed subtype were given a value of 1, otherwise a value of 0 was given. Thereafter, 100 genes for each histotype with statistically significant Odds ratios (*P* < 0.05) were selected for further analysis. Due to a slight overlap in the number of significant genes identified in more than one histotype (*i.e.* for HGSC vs all others and EC vs HGSC), a total of 393 genes were identified. These 393 genes were then used to assess the classification possibility of the histotypes. Random Forest (RF) was used as a classification tool, which requires the user to specify the number of genes that are randomly selected to grow each classification tree. To identify the ideal number of genes, grid search was used with Kappa statistics as an accuracy measurement. The search for statistically significant genes in the training cohort (n=95) was performed in repeated runs in the RF model with selection based on construction of trees of 1000 splits, each with 7 variables respectively. For further analysis, candidate biomarkers specific for each histotype with the highest fold change and variation in expression levels (arbitrary set level of raw RNA-seq counts >150), and commercial antibodies available were then selected for subsequent immunohistochemical analysis.

### Immunohistochemistry and data analysis

Tissue microarrays (TMA) were constructed using three 1.0 mm cores per tumor sample. Four micrometer FFPE sections (full-face and/or TMA slides) were prepared on Dako FLEX IHC microscope slides (Agilent Technologies) and dried in an oven for 1 hour at 60°C. Optimal antibody dilutions were achieved using an optimization panel consisting of 15 full-face FFPE ovarian carcinomas representing varying histotypes (HGSC, EC, MC, CCC) and International Federation of Gynecology and Obstetrics (FIGO) stages (**Supplementary Figure 1**). Immunostaining was performed for rabbit anti-TFF1 (Sigma-Aldrich HPA003425, 1:1000 dilution), rabbit anti-TFF2 (Sigma-Aldrich HPA036705, 1:100 dilution), and rabbit anti-TFF3 (Sigma-Aldrich HPA035464, 1:1000 dilution) on a Dako Autostainer Plus (Agilent Technologies) using Dako EnVision FLEX visualization systems. Full-face slides were used to perform initial IHC for TFF1 and TFF3 (n=206). TFF2 expression was scored using TMA samples (n=206). TFF1 and TFF3 expression were then scored on TMAs containing the MC and EC histotypes (n=103, including 29 MCs, 46 ECs and 28 of the 94 HGSC samples). Deparaffinization and antigen retrieval were performed using EnVision FLEX high pH target retrieval solution (pH 9). Staining and counterstaining were performed using liquid DAB (3,3′-diaminobenzidine) 2-component system and EnVision FLEX hematoxylin (link), respectively. After immunostaining, the sections were rinsed with deionized water, dehydrated in an ethanol series (comprised of 70, 95 and 100% ethanol), cleared in xylene and finally mounted. To facilitate histological assessment, FFPE sections were also stained with hematoxylin and eosin.

Analysis of the immunostained tissue sections was performed by independent board-certified pathologists (EWR and AK, blinded to histopathological and clinical data) using an Olympus BX45 light microscope. Assessment of tumor cell staining was executed with the modified histochemical score (H-score). By using the formula ([1 x %1+] + [2 x %2+] + [3 x %3+]), the semi-quantitative H-score (ranging from 0 to 300) was calculated based on the estimated percentage and intensity of positively stained tumor cells (no staining: 0 = negative, light yellow to yellow staining: 1+ = weak positive, light – medium brown staining: 2+ = moderate positive, and dark brown staining: 3+ = strong positive staining) (41). Cases containing less than 1% positively stained tumor cells were defined as negative. Full-face sections without representative tumor were excluded and another representative slide was selected. If one or more TMA punches were missing and/or if no tumor was present, the mean H-score was calculated. Although the intracellular distribution and sub-cellular staining compartment (*e.g.* cytoplasm, cell membrane, nucleus) was identified and recorded, only the intensity and percentage of immunostaining in the tumor population was included in the H-score. The TMAs were scanned (40x magnification) with a Leica SCN400 scanner and visualized using the Leica SCN400 Image Viewer software (v 2.2.0.3789) with up to 80x magnification.

### Statistical analysis

Statistical analyses were performed using a 0.05 p-value cutoff in R/Bioconductor (version 3.5.1). All p-values are two-sided. To compare expression levels for the TFF genes/proteins between the different histotypes, log2-transformed RNA-seq counts or H-score values were used to construct box plots using the ggpubr (version 0.2.1.999) R package (42) with the Kruskal-Wallis test and Benjamini-Hochberg adjusted p-values. Protein (H-score) and RNA expression levels (RNA-seq counts) were compared using log2-transformed expression values with a 95% confidence interval. To assess the relationship between TFF1, TFF2, and TFF3 protein expression patterns, pairwise Pearson correlation coefficients (interpretated according to Device Mapper statistics, *dmstat1* (43) weak correlation for *r* between 0 and 0.3 [0 and -0.3], moderate correlation for *r* between 0.3 and 0.7 [-0.3 and -0.7], and strong correlation for *r* between 0.7 and 1 [-0.7 and -1]) were calculated using the *corrplot* (version 0.88) R package (44–46), as well as *cor.test* R function and visualized for each protein pair using scatterplots with the *ggplot2* (version 3.3.3) R package (47).

## Results

### Random forest classification of RNA-seq data identifies candidate genes associated with the ovarian cancer mucinous invasive histotype

**Figure 1:**
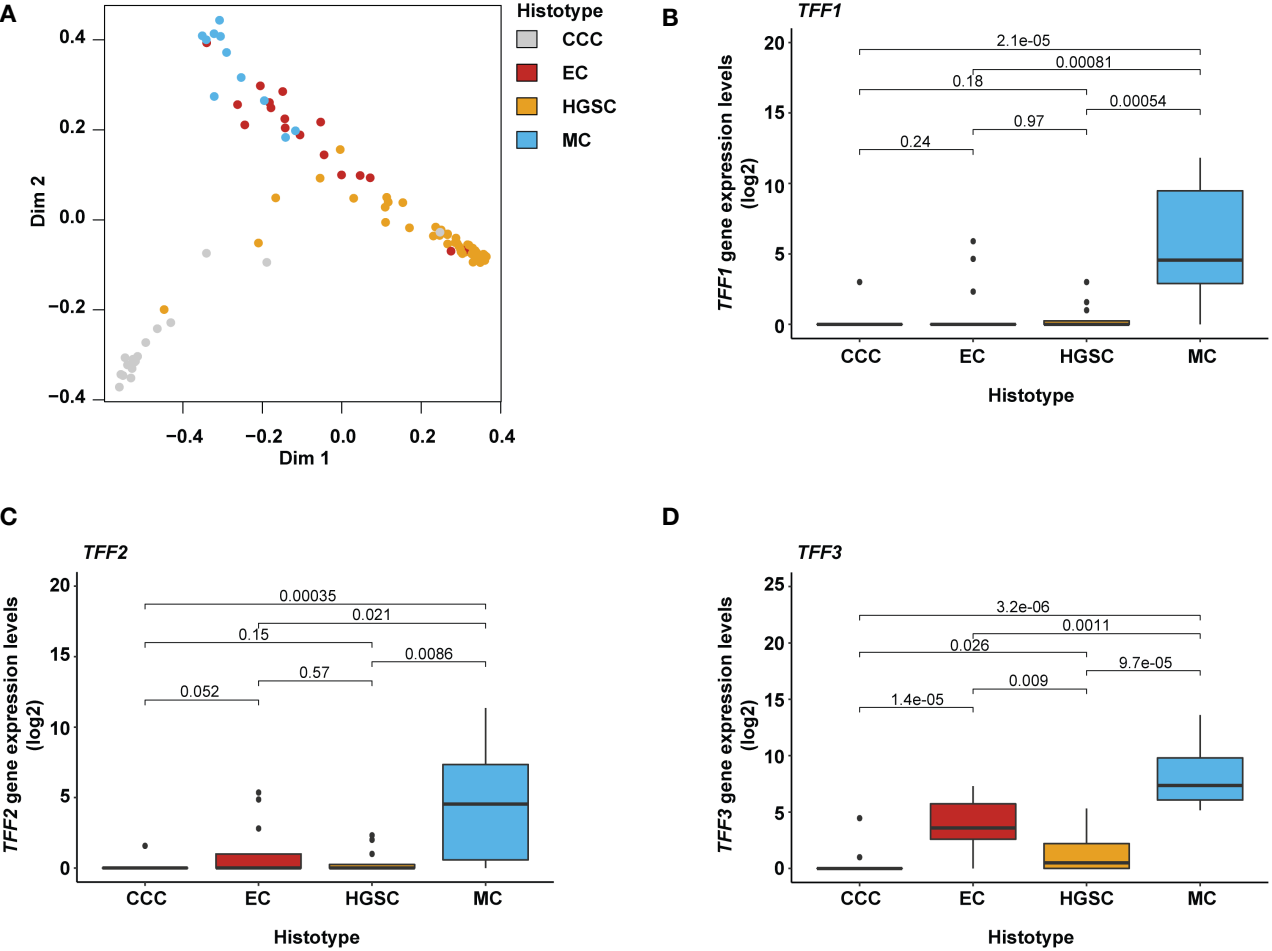
Gene-specific expression in mucinous ovarian cancer cell component. **(A)** MDS (multidimensional scaling) plot illustrating the specificity of the 393 identified genes classifying the different ovarian carcinoma histotypes. **(B–D)** Box plots showing the distribution of *TFF1*, *TFF2*, and *TFF3* gene expression levels in relation to the different histotypes with the Kruskal-Wallis test and Benjamini-Hochberg adjusted p-values. Expression levels were found to be elevated in MC ovarian carcinomas and small populations in the EC and HGSC histotypes; in EC samples showing mucinous differentiation (an attribute also seen in the HGSC histotype). *TFF1* and *TFF3* gene expression levels were shown to be significantly elevated for MC compared to other histotypes. *CCC, Clear cell carcinoma; EC, Endometroid carcinoma; HGSC, High-grade serous carcinoma; and MC, mucinous carcinoma*.

To identify gene expression patterns associated with specific OC histotypes (CCC, EC, HGSC, and MC), RNA sequencing data from our previous work (44) were re-evaluated using Random Forest (RF) classification. Consequently, the RF classification identified 393 genes for CCC (n = 98), EC (n = 97), HGSC (n = 103), and MC (n = 97) with a discriminating accuracy harboring an error rate of 11.6% (**Table 2**). The highest individual error rate was found for EC (6/17, 35%; **Figure 1A** and **Table 2**). Most of the misclassified EC samples (n = 5) were interpreted as HGSC. On the contrary, the majority of HGSCs were correctly matched (2% error rate).

**Table 2:** Confusion matrix demonstrating Random Forest classification of ovarian carcinoma histotypes using RNA sequencing data (training cohort, n=95).

Histotype	CCC (n = 17)	EC (n = 17)	HGSC (n = 50)	MC (n = 11)	Class Error Rate
**CCC**	**15**	0	2	0	0.12
**EC**	0	**11**	5	1	0.35
**HGSC**	1	0	**49**	0	0.02
**MC**	0	1	1	**9**	0.18

Bold numbers symbolize concordance. Horizontal: Histotypes as determined by histopathological evaluation. Vertical: RF classification. CCC, Clear cell carcinoma; EC, Endometroid carcinoma; HGSC, High-grade serous carcinoma; MC, Mucinous carcinoma.

Compared to the other histotypes (CCC, EC, and HGSC), the *TFF1* and *TFF3* genes were among the 20 top ranking protein-coding genes associated with the MC histotype (**Supplementary Table 1**) and thus selected for further analysis. As the TFF gene family is comprised of three protein-coding genes (*TFF1*, *TFF2*, and *TFF3*), *TFF2* was also included in the study despite not being represented among the 97 MC-associated genes. All three TFF genes revealed significantly higher gene expression levels in MC samples than the other histotypes (**Figures 1B–D**). However, expression in some MC samples was found to be very low (*i.e*. *TFF1* and *TFF2* RNA-seq raw counts less than 10) or not detected at all (*TFF1* and *TFF2*; **Supplementary Table 2A**). *TFF2* clearly exhibited the least MC-pure features by demonstrating both lower RNA-seq raw count values in general (*i.e.* five samples less than 10 RNA raw counts) as well as more non-MC samples with expression and, moreover, few MCs showing high *TFF2* expression patterns. The *TFF3* gene demonstrated comparably high expression levels in all MC samples. However, it was also frequently detected in samples of the EC subtype. In contrast, *TFF1* expression was, when present, in general more specific to MC. Additionally, the RNA sequencing analysis revealed several previously well-known human mucin-associated genes (*MUC2*, *MUC3A, MUC5AC*, *MUC13*, and *MUC17*) with expression in the MC histotypes samples (**Supplementary Tables 2A, B**).

### TFF protein validation by immunohistochemistry confirms TFF1 as a highly specific marker for MC

**Figure 2:**
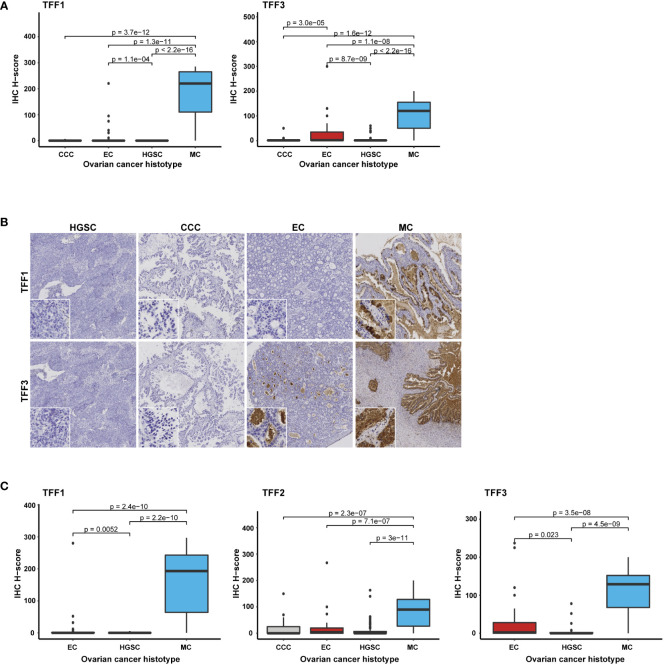
TFF1, TFF2, and TFF3 protein expression correlates with the MC histotype. Box plots illustrating protein expression in **(A)** full-face samples (n=206) for TFF1 and TFF3, **(B)** Immunohistochemical staining of TFF1 and TFF3 respectively presented in the different histotypes (to note the intraluminal secret sometimes was diffusely stained [TFF3], but left tumor cellular compartment negative), and **(C)** all three proteins (TFF1-TFF3) in TMA samples (n=103 for TFF1, n=102 for TFF3, and n=204 for TFF2) with the Kruskal-Wallis test and Benjamini-Hochberg adjusted p-values. *CCC, Clear cell carcinoma; EC, Endometroid carcinoma; HGSC, High-grade serous carcinoma; and MC, mucinous carcinoma*.

The immunohistochemical analysis of TFF1 and TFF3 protein expression showed positive staining patterns with predominance in the MC histotype (**Figures 2A–C** and **Table 3**). Specifically, TFF1 expression was even more specific for the MC histotype than TFF3, which was consistent with results from RNA seq. On average, TFF1 expression were significantly higher in MCs than the other histotypes in both full-face (TFF1 H-score in MC vs non-MC samples, mean: 179 vs 3, median: 220 vs 0, range: 0-285 vs 0-220; H-score values ≥100 in 76% [22/29] vs 0.6% [1/177]) and TMA samples (TFF1 H-score MC vs non-MC samples, mean: 156 vs 6; median: 193 vs 0; range: 0-297 vs 0-280; H-score values ≥100 in 62% [18/29] vs 1% [1/74]; **Figures 2A–C**, **Table 3**, **Supplementary Table 3**, and **Supplementary Figure 2**). Subsequently, TFF1 expression was focused to the MC histotype, frequently with moderate and high TFF1 H-score values (*i.e.* ≥50 and ≥100). Moderate and high TFF1 expression was otherwise only observed in a single non-MC sample (EC, sample 6193 from the validation cohort), which reached H-scores 220 and 280 in the TMA and full-face samples (**Table 3**). This sample exhibited cytoplasmic staining of variable intensity; however, goblet vacuoles were present and, hence, a sample including mucinous features. Unambiguous TFF1 expression was detected in a few additional EC samples (*i.e*. H-score ≥50 in the TMA- and/or full-face evaluation for 2 EC samples [H-score 52/90 and 32/75, respectively]). Notably however, only a few of the remaining ECs exceeded H-score level 10, leaving the vast majority as negative (80% of the 46 TMA samples and 85% of the 46 full-face samples). Of the 15 full-face ECs with any TFF1 positivity, mucinous features, including goblet vacuoles, was found in 3 samples, with cytoplasmic apical accentuation (features recorded from full-face slides) in an additional 2 samples.

**Table 3:** Frequency of protein expression in different OC (ovarian cancer) histotypes according to arbitrary H-score cut-off values.

Biomarker	CCC, n (%)	EC, n (%)		HGSC, n (%)		MC, n (%)
TFF1 (TMA), n=103	0	46	28		29	
H-score >1		9 (20)	0 (0)		26 (90)	
H-score ≥10		6 (13)	0 (0)		25 (86)	
H-score ≥50		2 (4)	0 (0)		24 (83)	
H-score ≥100		1 (2)	0 (0)		18 (62)	
TFF2 (TMA), n=204	37	46	93		28	
H-score >1	15 (41)	27 (59)	42 (45)		26 (93)	
H-score ≥10	10 (27)	17 (37)	18 (19)		25 (89)	
H-score ≥50	5 (13)	6 (13)	5 (5)		19 (68)	
H-score ≥100	1 (3%)	6 (13)	2 (2)		12 (43)	
TFF3 (TMA), n=102	0	46	28		28	
H-score >1		26 (57)	7 (25)		27 (96)	
H-score ≥10		15 (33)	3 (11)		26 (93)	
H-score ≥50		6 (13)	2 (7)		23 (82)	
H-score ≥100		4 (9)	0 (0)		19 (66)	
TFF1 (Full-face), n=206	37	46	94		29	
H-score >1	0 (0)	7 (15)	0		26 (90)	
H-score ≥10	0 (0)	6 (13)	0		25 (86)	
H-score ≥50	0 (0)	3 (7)	0		24 (83)	
H-score ≥100	0	1 (2)	0		22 (76)	
TFF3 (Full-face), n=206	37	46	94		29	
H-score >1	4 (11)	25 (54)	9 (10)		28 (97)	
H-score ≥10	2 (5)	20 (43)	5 (5)		28 (97)	
H-score ≥50	1 (3)	6 (13)	2 (2)		22 (76)	
H-score ≥100	0 (0)	3 (7%)	0 (0)		18 (62)	

TMA, tissue microarray; TFF1, trefoil factor 1; TFF2, trefoil factor 2; TFF3, trefoil factor 3; CCC, clear cell carcinoma; EC, endometroid carcinoma; HGSC, high grade serous carcinoma; MC, mucinous carcinoma; n, number.

TFF3 expression patterns were found to be quite similar to TFF1. However, the TFF3 protein was not exclusive to the MC histotype, but was also detected in several CCC, EC, and HGSC samples. In addition, although slightly more than half of the EC samples (26/46 TMA and 25/46 full-face) showed TFF3-positivity to some extent (*i.e.* H-score >1), the majority were of weak or moderate staining intensity (40/46 [87%] with H-scores ≤50, **Table 3**). TFF3 H-scores ≥100 was only found in 4 TMA/3 full-face scored EC samples. Consistent with TFF1, the strongest TFF3 expression was detected in EC sample 6193 (full-face H-score 300; 5 in TMA). In HGSC, moderate and cytoplasmic staining intensity with scoring values ≥ 50 was detected in a few (n=3) samples (#1: validation cohort sample 5679, H-score TMA: 78/full-face 2; #2: training cohort sample 5048, H-score TMA: N/A [not tested]/full-face 60; #3: training cohort sample 342, TMA 51/full-face 50). The remaining TFF3-positive non-MC histotype cases all had lower expression (*i.e.* H-score < 50; **Figures 2A–C**, **Table 3**, and **Supplementary Table 3**).

The TFF2 antibody was evaluated in 204/206 TMA hybridized samples (99%; 2 samples [MC sample 110 and HGSC sample 5353] were not assessed due to missing tumor and/or core N/A [not available]) and displayed a more heterogeneous staining pattern (**Figure 2B** and **Table 3**). Although all except two of the 28 MC samples exhibited positive staining to some extent (*i.e.* H-score >1, scoring range of all MC 0-300), TFF2 expression in the MC cohort was in general lower than TFF1 and TFF3 expression (*i.e.* H-score mean, H-score median both lower than 100 [mean: 97, median: 92, **Supplementary Table 3**]). Moreover, in the non-MC samples (n=176, H-score range 0-267) both moderate to intense TFF2 staining was not only more commonly observed but also intense in a few samples. Strong staining (*i.e.* H-score ≥ 100) was found in 9/176 (5%) samples and moderate (*i.e.* H-score ≥ 50) in 16/176 samples (9%; **Table 3**). Except ECs, high TFF2 expression levels were detected in one CCC (H-score level 150) and two HGSC samples (H-score level 163 and 140 respectively). This was contrasting to the expression patterns of TFF1 or TFF3 in the entire cohort (*i.e.* full-face assessment of all histotypes, n=206) which more clearly expressed the biomarkers in a) the MC cohort and b) ECs with mucinous features. TFF2 expression in the EC histotype was observed with moderate expression with the same frequency (6/46, 13%; **Table 3**) as TFF3. However, high TFF2 H-score levels from 100 were more common in 6 (13%) EC samples as well (H-score 100: n=3; 110: n=2; and 267: n=1, sample 6193). To summarize, TFF2 did not characterize the non-MCs by higher median or mean H-score values (mean: 14, median: 0,5, range: 0-267; **Supplementary Table 3**), but by displaying a higher number of CCCs and HGSCs with a higher range of expression. Additionally, fewer MCs presenting a convincing expression pattern, thereby symbolizing the biomarker with the least discrimination potential.

### Trefoil factor family members display intracytoplasmic staining patterns

Briefly, IHC assessment of the TMA slides revealed intracytoplasmic staining of the TFF1, TFF2 and TFF3 proteins. Additionally, TFF2 protein expression was also observed in the cell membrane. Nuclear protein positivity was rare and generally weak. TFF expression was most prevalent in the MC population, where protein staining was focused to the cytoplasm and intracytoplasmic vacuoles (including goblet cell morphology). When present, the vacuolar staining was generally more intense than other cytoplasmic components. Pure cell membrane or pure nuclear staining was not found in the MC cohort. An accentuated intracytoplasmic TFF1 and/or TFF3 staining pattern was found in a few non-MC samples, most frequently in the ECs (**Table 3**). As previously discussed, this was particularly found in ECs with mucinous differentiation including a few with vacuolar (goblet cell) morphology. Moreover, when stronger staining detected, an apical cellular localization was frequently found. The remaining EC samples showed a cytoplasmic or a non-specific combined cytoplasmic and membranous expression, mainly with hybridization with TFF3 and TFF2 (**Figures 2A, B**; **Table 3**). When present, the squamous component was negative.

More specifically, TFF3 nuclear staining was found in a few non-MC samples (n=10, **Supplementary Table 4**). Weak nuclear TFF2 IHC response (TMA only) was exclusively found in one CCC case. For TFF1, this pattern was not observed at all. Interestingly, TFF2 staining (H-score was frequently (78/204 samples) observed in the cell membrane, either in combination with (n=61) or without (n=17) cytoplasmic positivity. Besides one EC sample showing very weak membranous TFF1 staining, this IHC phenomena was primarily shown for TFF2 staining. Membranous involvement was, however, found in all histotypes specifically involving 14 CCCs, 13 ECs, 36 HGSCs, and 15 MCs (**Supplementary Table 5**). Exclusive membranous expression was commonly present in the CCC histotype, all with the glycogen rich, optic vacuolated clear cellular cytoplasmic morphology (8/14 samples, H-score range 3-85). In contrast, membranous positivity was always combined with cytoplasmic staining in the MC cohort. However, of the samples having selective membranous TFF2 positivity (H-score range 2-85), the corresponding RNA sequencing data available for 9 non-MC samples showed very low expression (*TFF2* RNA-seq count range 0-10, median: 0, mean: 1.6). In comparison, samples having protein expression levels exceeding H-score 10 in combination with low RNA-seq detection levels of *TFF1 or TFF3* counts (low as an arbitrary set level of RNA-seq count < 5) were rare, *i.e.* n=2 for TFF1 (n=2 EC samples [#1: EC H-score TMA 5/full-face 40, RNA counts 0; #2: EC H-score TMA 32/full-face N/A; RNA counts 5]) and n=2 also for TFF3 (n=2 non-MC, non-EC samples [1 CCC: H-score TMA N/A/full-face 50, RNA counts 0; 1 HGSC H-score TMA 26/full-face 0, RNA counts 1]), *N.B*. none of these cases with membranous positivity). Remarkably, no staining was detected in the corresponding full-face slides for the TFF3-expressing HGSCs.

### Concordance of IHC scoring on TMA and full-face samples

**Figure 3:**
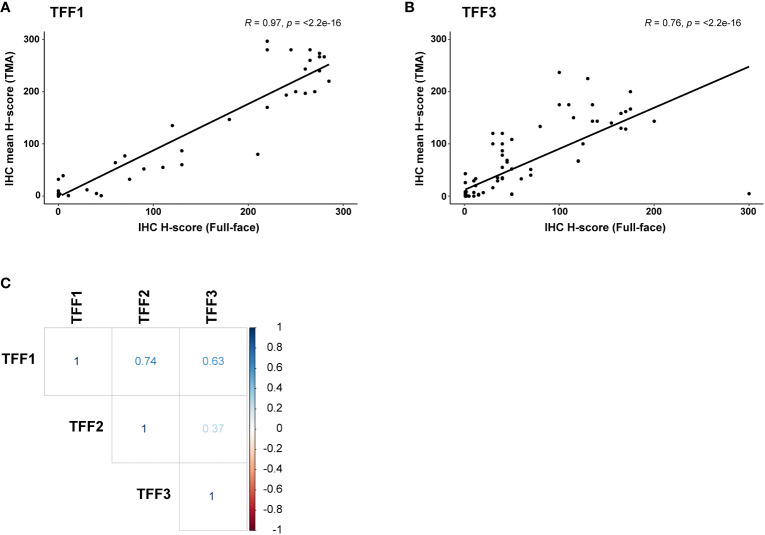
Comparison of TFF1, TFF2, and TFF3 protein expression in ovarian carcinoma samples (n=103) using pairwise Pearson correlation. Pearson correlation density plot comparing immunohistochemistry (IHC) scoring in full-face vs TMA samples for **(A)** TFF1 and **(B)** TFF3. **(C)** Using TMAs, TFF1 expression was found to be positively correlated with TFF2 (strong correlation, *r* > 0.7) and TFF3 (moderate correlation, *r* > 0.6) expression. No significant co-expression was found for TFF2 and TFF3.

Pearson correlation was then used to compare TFF1 and TFF3 protein expression patterns on the full-face sections and TMAs. This analysis revealed a strong correlation (*r* > 0.7) and high concordance between both sample types (**Figures 3A, B**). Moreover, pairwise Pearson correlation demonstrated the strongest correlation in protein expression patterns between TFF1 and TFF2 (*r* = 0.74), followed by TFF1 and TFF3 (*r* = 0.63; **Figure 3C**), thereby demonstrating frequent co-expression between TFF1 with both TFF2 and TFF3 (**Figure 3C**).

## Discussion

The present study aimed to identify novel histotype-specific biomarkers for early-stage OC using RNA sequencing and IHC technology. Of the 393 candidate genes, *TFF1* and *TFF3* gene expression was confirmed to stratify samples with mucinous differentiation and distinctly elevated *TFF1* and *TFF3* levels were found in the MC subtype exclusively. Moreover, expression was detected in ECs with mucinous features. While *TFF3* was frequently expressed regardless of histotype, *TFF1* was primarily expressed in MC. Validation of our RNA-seq results using IHC demonstrated concordance between RNA and protein expression patterns for TFF1, TFF2, and TFF3. Consequently, strong (H-score values ≥ 100) and moderate (H-score values ≥ 50) TFF1 and TFF3 was shown in most MC samples. Although some non-MC samples occasionally exhibited TFF protein positivity, very few reached staining intensities comparable to those found in the MC cohort. Further, we demonstrated high concordance in expression using full-face sections and TMAs, thereby justifying only examining TFF2 expression using TMAs. Despite displaying distinct protein expression patterns in ovarian carcinoma, Pearson correlation analysis revealed recurrent co-expression between TFF1/TFF2 and TFF1/TFF3.

Several biomarkers have been studied and implemented to facilitate the diagnostic evaluation of primary OC vs metastatic tumors, as well as a means of differentiating the epithelial OC histotypes (11, 19–23, 25, 30, 48–50). Herein, IHC analysis of the TFF-family was performed with antibodies that were validated in a TMA optimization panel consisting of four of the main OC histotypes (CCC, EC, MC, and HGSC) in tumor stages I-IV. In our study cohort, the specificity of the antibodies was thereby proven to agree with the algorithm-based ranking from RNA data. TFF1 specificity in mucinous ovarian cancer was previously explored using an *in silico* analysis of transcriptomics data (51). Moreover, our data are supported by gene expression analysis in studies where among several dysregulated genes, *TFF1* and *TFF3* have been upregulated exclusively in the MC histotype (31, 52, 53). Interestingly, hierarchical clustering of the expression profiles showed that the MC histotype and normal surface epithelium of the ovary clustered separately from all other OC histotypes. However, of all histotypes the highest number of dysregulated genes was found by comparing normal epithelium of the ovary and MC (52).

Previous studies have demonstrated the clinical value of the TFF protein family for ovarian carcinoma subtyping and immunohistochemistry algorithms to identify carcinomas of unknown origin (30, 31, 38). Identification of elevated TFF1 protein levels in diverse cancers such as pancreatic, colonic, and ovarian tumor tissues indicate a universal function in tumor progression *via* stimulation of cell migration, survival, invasiveness, and distant spread (36, 37). The *TFF3* gene has been shown to be an estrogen-regulated oncogene with prognostic value in estrogen-positive breast cancer and important contributor to gastric cancer progression (54). In addition, high *TFF3* levels are associated with poor survival, recurrence and distant metastasis in colorectal cancer (55). Elevated expression levels of *TFF1* and *TFF3* have been shown to correlate with changes in Ca125 (progression) and endocrine therapy response rates in ovarian cancer (56). Moreover, TMA-based studies demonstrated a correlation between elevated TFF3 expression, better prognosis, and lower recurrence rates in OC, but these studies were not stratified by histotype (54, 57). In a study by Kalloger et al. on OC histotyping, the *TFF3* gene was used to separate different epithelial ovarian cancer subtypes (31). The *TFF3* gene was further identified as highly expressed in the mucinous subtype of borderline ovarian tumors, suggesting specificity for mucinous differentiation (58).

Mucinous ovarian carcinoma is defined by large amounts of mucin in >90% of tumor cells (2, 3). In normal physiological conditions, mucins are large heavily glycosylated proteins secreted by epithelial cells constituting a physical barrier and providing protection and healing of epithelial tissues in the mucosal membranes. Few studies have explored the significance of the mucinous component in this cancer histotype. In the present study, the TFF-family genes were found to be up-regulated for MC. The TFF1 and TFF3 protein expression patterns were clearly connected to the presence of intracellular mucus or mucinous differentiation in epithelial cells. On the subcellular level, expression was localized to the intracytoplasmic compartment with/without the shape of intracytoplasmic mucin vacuoles and morphology of goblet cells, thereby enhancing the mucus component. Expression of TFF1, TFF2, and TFF3 was confirmed on the protein level with immunohistochemistry using antibodies validated by standardized protocols according to The Human Protein Atlas. Both TFF1 and TFF3 presented single peaks in protein arrays indicating an interaction with their antigens only (enhanced specificity), whereas the antibody for TFF2 passed specificity testing but was deemed to have low specificity. Only the TFF1 antibody had a single, distinct band using Western blot. Corresponding bands at predicted protein size of TFF2 and TFF3 were however not detected, hence defined as uncertain. Based on our results, the TFF2 protein demonstrated the poorest biomarker potential as non-specific, accentuated TFF2 staining was generally found in all histotypes. Membranous expression was also frequently found. Due to the demonstrated slight inferior specificity of the antibody, we suggest further testing in future studies before any final conclusions of both the usefulness and expression pattern can be ascertained.

In our study, several of the secreted genes of the well-studied MUC (mucin) gene family were discovered among the 97 identified MC-related genes. Of these, MUC2, MUC5A, and MUC6 have been found to be frequently co-expressed with TFF3, TFF1, and TFF2 (35). Moreover, the membrane bound mucin *MUC13* was co-expressed in all our mucinous samples. MUC13 has previously been found to have aberrant expression in ovarian cancer compared to normal ovarian tissue, especially in MC samples both compared to other histological OC types as well as adjacent normal tissues (59). True MC, as a rare disease, has a quite undefined origin and not fully understood molecular background (6, 10). Lack of normal mucin-secreting cells in the ovary challenges the elucidation of the cell of origin. Previous findings indicate that MC development is a multistep process with similarities to tumor development in colorectal carcinoma (52). Using GWAS analysis, Kelemen and colleagues identified specific MC histotype associated gene susceptibility loci and SNPs detected in other OC histotypes, which implies common features in the OC biological background (60).

Previous studies have implemented useful immuno-histochemical algorithms with antibodies to distinguish between ovarian carcinoma histotypes with 90% accuracy (*e.g.* the 8-protein panel p53, p16, PR, WT1, ARID1A, HNF1B, VIM, and TFF3) (20, 22, 23, 25, 26, 30, 49). Nevertheless, routine histology should still be used as a baseline to determine a final diagnosis. For ambiguous cases, immunohistochemistry can be helpful as a diagnostic tool. The possibility of using specific proteins as serum biomarkers, circulating tumor cells (CTCs) or liquid biopsies has recently been successfully developed for several cancer types. Both *TFF1* and *TFF3* mRNA have been demonstrated to be elevated in patients with metastatic breast cancer (61). Moreover, high serum levels of TFF3 in gastrointestinal cancer correlated with more advanced disease and poor therapeutic response (54, 55). In breast tumor tissue, *TFF1* and *TFF3* expression levels were inversely related to proliferation index (Ki67) and tumor grade. Expression of *TFF3* was more related to malignant tumors compared to the presence of *TFF1.* A correlation with *TFF2* levels was not found (62). In comparison with other histotypes, the CA125 (MUC16) serum-marker is known to be a relatively weak predictor of relapse for MCs. Use of a new biomarker in combination with a well-known serum-marker *e.g.* Ca125 (MUC16) or HE4, was suggested for REG4, a protein with mucin-like staining patterns which was studied in OC with mucinous differentiation by Lehetinen et al. in 2015 (51). Therefore, the TFF1 and TFF3 biomarkers may provide healthcare professionals with tools to better subclassify OC by histotype, more efficiently predict patient clinical outcome, and improve the treatment decision-making process for ovarian cancer.

A recent study showed that the CK7 (*KRT7*), CK20 (*KRT20*), and SATB2 immunohistochemical markers could assist in differentiating primary MCs from metastatic tumors (49). Although the present study could have been improved by including one or more of these markers, especially on the protein level, only *KRT20* was identified among the MC-associated genes based on our RNA-seq data. However, *KRT7* were present in most samples with expression levels exceeding RNA-seq count levels of 100 (all histotypes) and in some cases exceeding or reaching levels of 1000. In comparison, in our cohort *SATB2* results diverged having three-digit values in a minority of samples (all histotypes) but also, for both HGSCs, ECs as well as MCs, exhibiting single samples with contrastingly very high expression. The significance of this variation has not been further investigated. Moreover, the patients included in the study were diagnosed with ovarian carcinoma between 1993 and 2006, thus imposing the risk of suboptimal conditions for immunohistochemistry using FFPE samples (*e.g.* time of fixation, differences in batches and chemical conditions for tissue preparation, storage, etc.). Nevertheless, we found high concordance between our RNA (fresh-frozen tissue samples) and protein (FFPE samples) data. The present study has a number of other limitations, including small sample size, particularly for each specific histotype, and general immunopositivity of the TFF proteins in the mucinous component (*e.g.* in ECs), which might reduce the potential of the TFF-family to be used as sole discriminator of the MC histotype. The included patients were operated for their ovarian cancer between 1993 and 2006, before implementation of the first national guidelines for ovarian cancer in Sweden (2012), in which appendectomies were routinely carried out when removing an ovarian cancer of suspected or confirmed mucinous origin. To minimize confusion with metastatic events, complete clinical data, macro- and microscopic evaluation of the tumor, as well as the combination of immunohistochemistry and gene expression data is preferable. However, the medical records for the MC cohort were also scrutinized for the presence of meta- and/or synchronous tumors (especially gastro-pancreatic-intestinal), the intraoperative status of the abdominal cavity, and any data for the appendix (previously/simultaneously performed appendectomy was available for 5 patients). Therefore, future studies should be performed using larger cohorts using both gene expression and IHC data for known discriminating markers to further minimize the risk that metastatic events are included and validate TFF1 and TFF3 expression as stratifiers of different OC histotypes (26, 52, 60).

The TFF protein family has previously been linked to tumor malignancy, progression, and classification. In the present study, we demonstrate overexpression of the *TFF1* and *TFF3* genes in early-stage ovarian MC histotype, with mainly intracytoplasmic localization. For the individual patient, prognosis and disease-free survival rely on a correct and early diagnosis (6). The identification of biomarkers for early OC disease could lead to more patients being diagnosed at an early stage and use of histological subtyping for precision medicine. This, in turn, may result in improved patient survival (1, 6, 63–65). The nature of a study population (OC stage I-II) enables a reflection of the TFF-family in a perspective of early events of malignant transformation. Future studies should address the role of the TFF protein family both in tumor progression by analyzing expression in late stage (stage III and IV) OC, as well as more deeply investigate their presence in other malignant tumors. This in turn would allow us to elucidate their potential role as disease-specific biomarkers, enabling their inclusion in established IHC algorithms for ovarian carcinoma histotyping as well as tool to monitor an already diagnosed patient with OC disease.

## Data availability statement

The datasets presented in this study can be found in online repositories. The names of the repository/repositories and accession number(s) can be found in the article/**Supplementary Material**.

## Ethics statement

The studies involving human participants were reviewed and approved by Regional Ethical Review Board. Written informed consent for participation was not required for this study in accordance with the national legislation and the institutional requirements.

## Author contributions

KH and TP were responsible for overall study concept, design of experiments. PDK and PK collected the clinical data. EWR, TP and SN performed the correlation of the gene expression data with different histotypes for study gene selection. DP performed the IHC experiments. EWR and AK pathologically reclassified the histotypes and performed the microscopic evaluation of hybridized tissue sections. EWR and TP performed the statistical analyses. PDK and PK provided clinical information and support related to ovarian carcinoma. EWR, KH and TP were responsible for planning and preparation of the IHC experiments, data analysis and manuscript writing. All authors contributed to the article and approved the submitted version.
