# Supplementary figures and images for: Trefoil factor family proteins as potential diagnostic markers for mucinous invasive ovarian carcinoma
Source: Front Oncol. 2023 Feb 2;12:1112152. doi: 10.3389/fonc.2022.1112152 (PMC9932968; doi:10.3389/fonc.2022.1112152)

**A**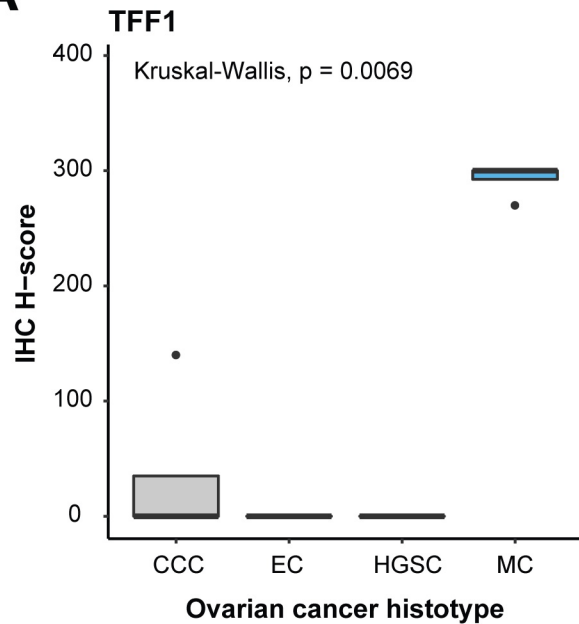**B**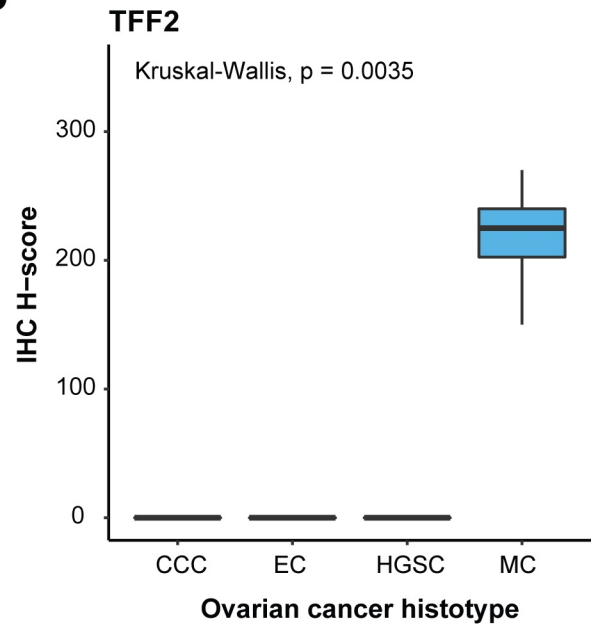**C**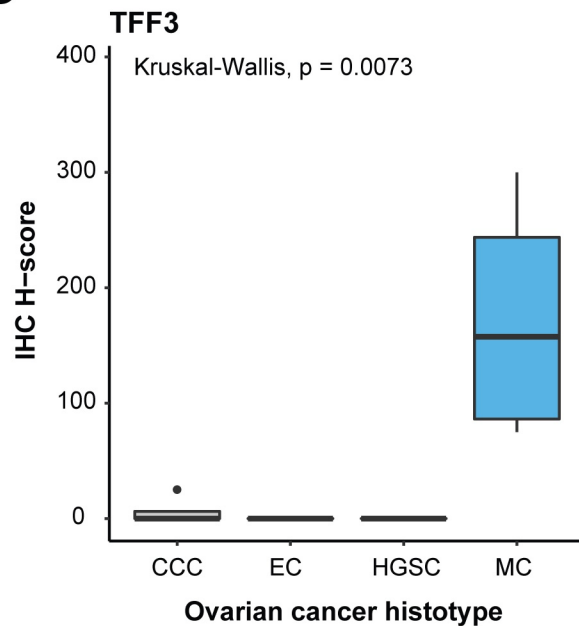

Supplement: Supplementary file 1 [file DataSheet_1.pdf]

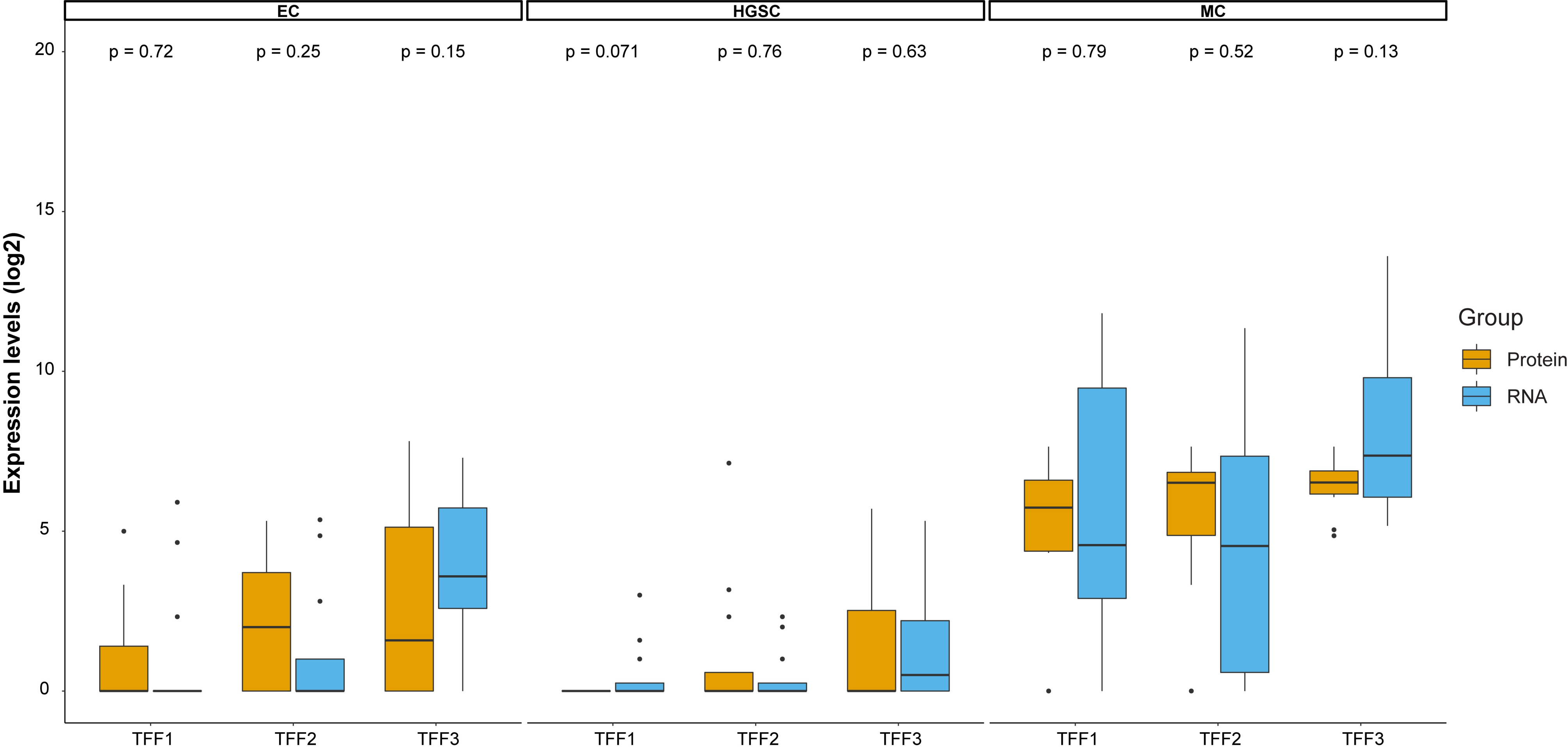

Supplement: Supplementary file 2 [file DataSheet_2.pdf]
